# Composable security of CV-MDI-QKD with secret key rate and data processing

**DOI:** 10.1038/s41598-023-37699-5

**Published:** 2023-07-19

**Authors:** Panagiotis Papanastasiou, Alexander G. Mountogiannakis, Stefano Pirandola

**Affiliations:** Department of Computer Science, University of York, York, YO10 5GH UK

**Keywords:** Quantum information, Quantum optics, Computer science

## Abstract

We provide a rigorous security proof of continuous-variable measurement-device-independent quantum key distribution which incorporates finite-size effects and composable terms. In order to use realistic and optimized parameters and be able to derive results close to experimental expectations, we run protocol simulations supported by a Python library, including all the protocol operations, from simulating the quantum communication till the extraction of the final key.

## Introduction

Quantum key distribution (QKD) uses a quantum channel for the transmission of signals between two distant legitimate parties to create a shared secret key^1–3^. The secret key can be later utilized for the symmetric encryption of confidential messages exchanged between the parties. In particular, based on the laws of quantum mechanics, QKD allows for the detection of the presence of an eavesdropper in the quantum channel and the quantification of the compromised amount of information^4,5^. Depending on this amount, the shared data after the quantum communication can be compressed into a shorter shared key, about which the eavesdropper possesses negligible knowledge. This leads to quantum-safe applications, i.e., applications safe against attacks by large quantum computers.

In the beginning, QKD protocols were based on a discrete-variable (DV) encoding^6–8^, such as the polarization of a photon. The security of such protocols has been thoroughly investigated. More recently, protocols that exploit continuous degrees of freedom, such as the position and momentum of the electromagnetic field^9^ have been developed^10–12^. These are called “continuous-variable” (CV) QKD protocols. CV-QKD is highly compatible with the current telecommunications and, consequently, promises simpler and cost-effective practical implementations. Furthermore, it produces high rates, which approach the capacity limit of repeaterless quantum communications, also known as PLOB bound^13^. Their performance with respect to larger distances has improved significantly^14,15^.

Crucial improvements have also been demonstrated in their security level. We have different levels of security (on top of the levels listed below, the security is characterized also by the level of attacks, i.e, individual, collective, or coherent ones^9^) according to the assumptions taken into consideration when one calculates the secret key rate (secret bits per channel use). The first is the asymptotic security which assumes an infinite number of signals. The finite-size security^16^ refers to the practical use of a finite number of signals. And finally, the composable framework^17^ considers all the post-processing subroutines in the evaluation of the security of the protocol.

A standard QKD protocol provides security against channel attacks, where the eavesdropper interacts with the quantum signals propagating through the channel. However, equivalently crucial, if not more dangerous, are the attacks connected with the preparation or detection processes, where the eavesdropper has direct access to the labs of the two legitimate parties. These attacks are known as side-channel attacks^1^. MDI-QKD^18,19^ and CV-MDI-QKD^20–24^ have the intrinsic property of relieving the parties from any detection obligation. In fact, it uses an intermediate relay, which is responsible for the detection part of the protocol. The relay can be considered part of the channel, i.e., under the control of the eavesdropper. The outcomes of this detection are classically broadcast to the parties, who utilize them to build correlations between their data strings. This configuration can be used as a basis for constructing multi-user applications^25,26^ (see^27^, Appendix VII) that can be extended to QKD networks^28^. Experimental implementations have also taken place recently^29,30^.

Here, we focus on a simulation analysis similar to Ref.^31,32^ but for the CV-MDI protocol^20,21^. Its finite-size security analysis has been presented in Ref.^33^, with a first composable study discussed in Ref.^34^. In “Protocol and asymptotic secret key rate” section, we give a detailed summary of the protocol and the calculation of its asymptotic key rate. Then, we assume finite-size effects and describe the postprocessing subroutines. In “Composable security” section, we adapt the composable proof of Ref.^17^ to the CV-MDI protocol. This proof removes an issue from a previous treatment^34^ (see also^27^, Appendix VI). In “Privacy amplification” section, we explain how the parties apply the appropriate amount of compression to the data to extract a secret key, according to the previous analysis. We present all the results of the simulation with the help of a developed Python library in “Simulation and results” section. Conclusions are in “Conclusion” section.

## Security analysis

In this section, we investigate the quantum communication part of the protocol (considering the classical postprocessing part ideal). We focus on the potential to build strong correlations secret to the eavesdropper considering an infinite number of signals between the parties. We present this analysis here because, as we show later, the asymptotic secret key rate is an integral part of the composable secret key rate, along with the correction terms because of the non-ideal character of the classical postprocessing procedures.

### Protocol and asymptotic secret key rate

Alice and Bob prepare coherent states $|\alpha \rangle$ and $|\beta \rangle$ with amplitudes $\alpha =(1/2)(Q_A+\textrm{i}P_A)$ and $\beta =(1/2)(Q_B+\textrm{i}P_B)$, carried by modes *A* and *B* respectively. In particular, they encode the real vectorial variables $\varvec{\alpha }=(Q_A,P_A)$ and $\varvec{\beta }=(Q_B,P_B)$ following the Gaussian distributions

1 $$\begin{aligned} G(\varvec{\alpha })&=\frac{\exp \big [-\frac{1}{2}(Q_A^2+P_A^2)/\sigma ^2_A\big ]}{2\pi \sigma ^2_A}, \end{aligned}$$

2 $$\begin{aligned} G(\varvec{\beta })&=\frac{\exp \big [-\frac{1}{2}(Q_B^2+P_B^2)/\sigma ^2_B\big ]}{2\pi \sigma ^2_B}, \end{aligned}$$

with variances $\sigma _A^2$ and $\sigma _B^2$ respectively. The two bosonic modes travel to an intermediate relay, where a Bell measurement is applied to them with outcome $\gamma =(1/2)(Q_R+\textrm{i}P_R)$. We also use the notation $\varvec{\gamma }=(Q_R,P_R)$.

**Figure 1:**
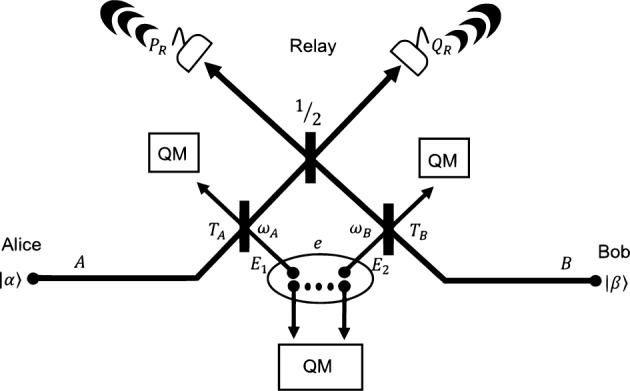
Alice and Bob send coherent states $|\alpha \rangle$ and $|\beta \rangle$ with modes *A* and *B* to the intermediate relay. Eve’s modes $E_1$ and $E_2$ interact with the traveling modes via beam splitters with transmissivities $T_A$ and $T_B$ respectively. Eve’s two-mode attack is characterized by thermal noise parameters $\omega _1$ and $\omega _2$ (see Eq. 3). Eve’s modes are stored in a quantum memory waiting for an optimal measurement after the communication between the parties.

Eve interacts with the traveling modes via a two-mode attack where mode $E_1$ is mixed with mode *A* through a beam splitter with transmissivity $T_A$ and mode $E_2$ with mode *B* through a beam splitter with transmissivity $T_B$ (see Fig. 1). The CM of Eve’s modes is given by

3 $$\begin{aligned} \textbf{V}_{E_1E_2}=\begin{pmatrix} \omega _A \textbf{I}&{}\textbf{G}\\ \textbf{G}&{} \omega _B \textbf{I} \end{pmatrix},~~\textbf{G}=\begin{pmatrix} g&{}0\\ 0&{} g^\prime \end{pmatrix}, \end{aligned}$$

where the bona fide conditions for *g* and $g^{\prime }$ are given in Ref.^21^. In fact, given the previous description (These attacks are collective Gaussian two mode attacks and represent the entangling cloner attack^35,36^ counterpart of a channel comprised of two links.), the best attacks are those with $g<0$ and $g^\prime >0$. Taking into consideration this area of values, one can see that as |*g*| and $|g^\prime |$ become larger, the modes become more quickly and more strongly correlated (entangled). Then, one can choose $g_\text {max}=\text {max}\{|g|,|g^\prime |\}$ and assume the attack with

4 $$\begin{aligned} \textbf{G}_\text {max}=\begin{pmatrix} -g_\text {max}&{}0\\ 0&{} g_\text {max} \end{pmatrix}, \end{aligned}$$

as the worst-case scenario. In such a case, the quadratures can be treated equivalently, as they follow the same probability distribution.

The outputs $Q_R$ and $P_R$ are dependent on the variables $Q_A$, $P_A$ and $Q_B$, $P_B$ according to the following equations:

5 $$\begin{aligned} Q_R=&\tau _B Q_B-\tau _A Q_A+Q_z, \end{aligned}$$

6 $$\begin{aligned} P_R=&\tau _B P_B+\tau _A P_A+P_z, \end{aligned}$$

where $\tau _A$ and $\tau _B$ are rescaling parameters connected to the overall attenuation via

7 $$\begin{aligned} \tau _A=&\sqrt{\eta _\text {eff} T_A/2}, \end{aligned}$$

8 $$\begin{aligned} \tau _B=&\sqrt{\eta _\text {eff} T_B/2}, \end{aligned}$$

and the noise variables $Q_z$ and $P_z$ have variance $\sigma _{z}^2$ such that

9 $$\begin{aligned} \sigma ^2_{z}=&\Xi +v_\text {el}+1, \end{aligned}$$

where $\eta _\text {eff}$ and $v_\text {el}$ are the calibrated detection efficiency and electronic noise of the detectors respectively. In Supplementary Appendix V, we show the details of the calibrated noise attack and its connection to the uncalibrated one (see Supplementary Appendix IV). Subsequently, we obtain

10 $$\begin{aligned} \Xi&=\frac{\eta _\text {eff}}{2}\bigg ((1-T_A)(\omega _A-1)+(1-T_B)(\omega _B-1)\bigg )\nonumber \\&\quad + \eta _\text {eff} g_\text {max} \sqrt{(1-T_A)(1-T_B)}, \end{aligned}$$

with

11 $$\begin{aligned} g_\text {max}=\max \{\sqrt{(\omega _A-1)(\omega _B+1)},\sqrt{(\omega _B-1)(\omega _A+1)}\}. \end{aligned}$$

In the EB representation, one introduces additional modes *a* and *b* in two-mode squeezed-vacuum (TMSV) states with modes *A* and *B*, respectively. These states have variances $\mu _A=\sigma _A^2+1$ and $\mu _B=\sigma _B^2+1$, respectively. Then, the encoding process is simulated by a heterodyne measurement on modes *a* and *b* with corresponding measurement outcomes $\tilde{\varvec{\alpha }}$ and $\tilde{\varvec{\beta }}$. The initial CM of the systems is given by

12 $$\begin{aligned} \textbf{V}_{aABbE_1E_2}=\textbf{V}_{aA}\oplus \textbf{V}_{Bb}\oplus \textbf{V}_{E_1E_2}, \end{aligned}$$

with $\textbf{V}_{aA}(\mu _A)$ and $\textbf{V}_{Bb}(\mu _B)$ being CMs of a TMSV state

13 $$\begin{aligned} \textbf{V}_\text {TMSV}(\mu )=\begin{pmatrix} \mu \textbf{I}&{}\sqrt{\mu ^2-1}\textbf{Z}\\ \sqrt{\mu ^2-1}\textbf{Z}&{}\mu \textbf{I} \end{pmatrix}, \end{aligned}$$

and $\textbf{Z}=\text {diag}\{1,-1\}$. The attack corresponds to applying a beam splitter with transmissivity $T_A$ between the modes *A* and $E_1$ and a beam splitter of transmissivity $T_B$ between modes *B* and $E_2$. The beam splitter symplectic operation with transmissivity *T* is given by

14 $$\begin{aligned} {\mathcal {B}}{\mathcal {S}}(T)=\begin{pmatrix} \sqrt{T}\textbf{I}&{}\sqrt{1-T}\textbf{I}\\ -\sqrt{1-T}\textbf{I}&{}\sqrt{T}\textbf{I} \end{pmatrix}. \end{aligned}$$

After the beam splitters, Alice’s and Bob’s modes $A'$ and $B'$ are mixed in a balanced beam splitter (i.e., $T=1/2$) and conjugate homodyne measurements are applied to the output modes with outcomes grouped in the variable $\varvec{\gamma }$. In fact, starting from a CM with the following general form

15 $$\begin{aligned} \textbf{V}_{FM}=\begin{pmatrix} \textbf{F}&{}\textbf{C}\\ \textbf{C}^\textsf{T}&{}\textbf{M} \end{pmatrix}, \end{aligned}$$

if we apply a homodyne measurement to mode *M* with outcome $x_M$, the CM after the measurement will be given by

16 $$\begin{aligned} \textbf{V}_{F|x_M}=\textbf{F}-\textbf{C} (\varvec{\Pi }\textbf{M}\varvec{\Pi })^{-1}\textbf{C}^{\textsf{T}}, \end{aligned}$$

with $\varvec{\Pi }={{\,\textrm{diag}\,}}\{1,0\}$ ($\varvec{\Pi }={{\,\textrm{diag}\,}}\{0,1\}$) for a *Q*(*P*)-measurement and $(.)^{-1}$ being the pseudo-inverse operation.

In this description, the CM after the relay measurements is given by

17 $$\begin{aligned} \textbf{V}_{ab|\varvec{\gamma }} =\begin{pmatrix} \phi \textbf{I}&{}\eta \textbf{Z}\\ \eta \textbf{Z} &{}\theta \textbf{I}\\ \end{pmatrix}, \end{aligned}$$

where

18 $$\begin{aligned} \phi ~=&~1+\sigma ^2_A-\frac{\tau ^2_A\sigma ^2_A(\sigma ^2_A+2)}{\tau _B^2\sigma ^2_B+\tau ^2_A\sigma ^2_A+\sigma _z^2}, \end{aligned}$$

19 $$\begin{aligned} \theta ~=&~1+\sigma ^2_B-\frac{\tau ^2_B\sigma ^2_B(\sigma ^2_B+2)}{\tau _B^2\sigma ^2_B+\tau ^2_A\sigma ^2_A+\sigma _z^2}, \end{aligned}$$

20 $$\begin{aligned} \eta ~=&~\frac{\tau _A\tau _B\sqrt{\sigma ^2_A(\sigma ^2_A+2)\sigma ^2_B(\sigma ^2_B+2)}}{\tau _B^2\sigma ^2_B+\tau ^2_A\sigma ^2_A+\sigma _z^2}. \end{aligned}$$

The conditional CM after the heterodyne measurement of mode b with outcome $\tilde{\varvec{\beta }}$ is given by

21 $$\begin{aligned} \textbf{V}_{a|\varvec{\gamma }\tilde{\varvec{\beta }}}=\left( \phi -\frac{\eta ^2}{\theta +1}\right) \textbf{I}. \end{aligned}$$

From the matrices

22 $$\begin{aligned} \textbf{V}_{a|\varvec{\gamma }}=\phi \textbf{I}, \end{aligned}$$

and $\textbf{V}_{a|\varvec{\gamma }\tilde{\varvec{\beta }}}$, we can calculate the mutual information between $\tilde{\varvec{\beta }}$ and Alice’s outcome $\tilde{\varvec{\alpha }}$ which is

23 $$\begin{aligned}I(\varvec{\tilde{\alpha }}:\varvec{\tilde{\beta }}|\gamma )=\frac{1}{2}\log _2\frac{\det \textbf{V}_{a|\varvec{\gamma }}+\textrm{tr}\,\textbf{V}_{a|\varvec{\gamma }}+1}{\det \textbf{V}_{a|\varvec{\gamma }\tilde{\varvec{\beta }}}+\textrm{tr}\,\textbf{V}_{a|\varvec{\gamma }\tilde{\varvec{\beta }}}+1}. \end{aligned}$$

One may also calculate, from the CM in Eq. (17), Eve’s Holevo information

24 $$\begin{aligned} \chi (E:\tilde{\varvec{\beta }}|\varvec{\gamma })&=H(E|\varvec{\gamma })-H(E|\tilde{\varvec{\beta }}\varvec{\gamma }), \end{aligned}$$

25 $$\begin{aligned}&=\int p(\varvec{\gamma })H(\rho _{E|\varvec{\gamma }})~\textrm{d}^2\varvec{\gamma }\nonumber \\&\quad -\int p(\tilde{\varvec{\beta }},\varvec{\gamma })H(\rho _{E|\tilde{\varvec{\beta }}\varvec{\gamma }})~\textrm{d}^2\varvec{\gamma }~\textrm{d}^2\tilde{\varvec{\beta }}, \end{aligned}$$

26 $$\begin{aligned}&=H(\rho _{ab|\varvec{\gamma }})-H(\rho _{a|\tilde{\varvec{\beta }}\varvec{\gamma }}), \end{aligned}$$

which is expressed in terms of conditional von Neumann entropies. Then by the assumption that Eve’s systems $E=E_1'E_2'e$ purify the whole output state, we have that the von Neumann entropy of the state $\rho _{E|\varvec{\gamma }}$ equals that of $\rho _{ab|\varvec{\gamma }}$, and similar equivalence holds between $\rho _{E|\tilde{\varvec{\beta }}\varvec{\gamma }}$ and $\rho _{a|\tilde{\varvec{\beta }}\varvec{\gamma }}$. These entropies are not dependent on the outcomes $\tilde{\varvec{\beta }}$ and $\varvec{\gamma }$ and can be expressed in terms of the symplectic eigenspectra $\{\nu _{\pm }\}$ and $\tilde{\nu }$ of of the CMs $\textbf{V}_{ab|\varvec{\gamma }}$ and $\textbf{V}_{a|\tilde{\varvec{\beta }}\varvec{\gamma }}$ respectively, so that

27 $$\begin{aligned} H(\rho _{ab|\varvec{\gamma }})-H(\rho _{a|\tilde{\varvec{\beta }}\varvec{\gamma }})=h(\nu _{+})+h(\nu _{-})-h(\tilde{\nu }), \end{aligned}$$

with

28 $$\begin{aligned} h(\nu ):=\frac{\nu +1}{2}\log _{2}\frac{\nu +1}{2}-\frac{\nu -1}{2}\log _{2} \frac{\nu -1}{2}. \end{aligned}$$

In terms of mutual information, the measurement variables $\varvec{\tilde{\alpha }}$ and $\varvec{\tilde{\beta }}$ in the EB scheme are equivalent to the rescaled P&M variables, $\varvec{\alpha }$ and $\varvec{\beta }$. Then the conditioning on $\varvec{\gamma }$ is equivalent to a displacement on the variables $\varvec{\alpha }$ and $\varvec{\beta }$ so that key-extraction variables, $\textbf{x}=(Q_x,P_x)$ and $\textbf{y}=(Q_y,P_y)$, need to be suitably constructed. In fact, the parties use the following relations

29 $$\begin{aligned} Q_x&=Q_A-u_Q Q_R , \end{aligned}$$

30 $$\begin{aligned} P_x&=P_A-u_P P_R, \end{aligned}$$

31 $$\begin{aligned} Q_y&=Q_B-v_Q Q_R, \end{aligned}$$

32 $$\begin{aligned} P_y&=P_B-v_P P_R . \end{aligned}$$

An optimal option for the parameters *u* and *v* is given by assuming a minimal correlation between the new variables, $\textbf{x}$ and $\textbf{y}$, and the relay outputs. This is explained by the fact that Eve should know as less as possible about $\textbf{x}$ and $\textbf{y}$ by knowing $\varvec{\gamma }$. Therefore, we impose

33 $$\begin{aligned} \langle Q_y Q_R\rangle =\langle Q_x Q_R\rangle =0,~~~\langle P_y P_R\rangle =\langle P_x P_R\rangle =0, \end{aligned}$$

so to obtain (These are the regression coefficients. Given a bipartition of a multivariate Gaussian distribution $\{\textbf{x}_1,\textbf{x}_2\}$ with CM $\varvec{\Sigma }$, the regression coefficients are given by the matrix $\varvec{\Sigma }_{12}\varvec{\Sigma }_{22}^{-1}$. One may write that $\textbf{y}=\textbf{x}_1|\textbf{x}_2=\textbf{x}_1-\varvec{\Sigma }_{12}\varvec{\Sigma }_{22}^{-1}\textbf{x}_2$.)

34 $$\begin{aligned} u_Q&=\frac{\langle Q_A Q_R\rangle }{\langle Q_R^2\rangle }=\frac{-\tau _A\sigma ^2_A}{\tau _B^2\sigma ^2_B+\tau ^2_A\sigma ^2_A+\sigma _z^2}, \end{aligned}$$

35 $$\begin{aligned} u_P&=\frac{\langle P_A P_R\rangle }{\langle P_R^2\rangle }=-u_Q, \end{aligned}$$

36 $$\begin{aligned} v_Q&=\frac{\langle Q_B Q_R\rangle }{\langle Q_R^2\rangle }=\frac{\tau _B\sigma ^2_B}{\tau _B^2\sigma ^2_B+\tau ^2_A\sigma ^2_A+\sigma _z^2}, \end{aligned}$$

37 $$\begin{aligned} v_P&=\frac{\langle P_B P_R\rangle }{\langle P_R^2\rangle }=v_Q. \end{aligned}$$

Therefore, one may write

38 $$\begin{aligned} I(\textbf{x}:\textbf{y})=I(\varvec{\alpha }:\varvec{\beta }|\varvec{\gamma })=I(\tilde{\varvec{\alpha }}:\tilde{\varvec{\beta }}|\varvec{\gamma }), \end{aligned}$$

where the first equality is proven in Ref.^27^, Appendix I.

The quantum mutual information between Eve’s system $E=E^\prime _1E^\prime _2e$ and Bob’s key-extraction variable $\textbf{y}$ when she has access to the variable $\varvec{\gamma }$ is given by^37^, Lemma 7.4.439and it is equal to the Holevo information $\chi (E:\textbf{y}|\varvec{\gamma })$ since $\textbf{y}$ is a classical variable. In particular, we have that, given $\varvec{\gamma }$, there is a function $\textbf{y}=f(\varvec{\beta })$ determined by the relations in Eqs. (31) and (32) such that $\varvec{\beta }=f^{-1}(\textbf{y})$. This allows us to apply the data processing inequality in both directions with respect to *y* and $\beta$ and obtain

40 $$\begin{aligned} \chi (E:\textbf{y}|\varvec{\gamma })=\chi (E:\varvec{\beta }|\varvec{\gamma }). \end{aligned}$$

At this point, one may define the asymptotic key rate

41 $$\begin{aligned} R_{\text {asy}}&=\zeta I(\textbf{x}:\textbf{y})-\chi (E:\textbf{y}|\varvec{\gamma }), \end{aligned}$$

42 $$\begin{aligned}&=\zeta I(\varvec{\alpha }:\varvec{\beta }|\varvec{\gamma })-\chi (E:\varvec{\beta }|\varvec{\gamma }), \end{aligned}$$

43 $$\begin{aligned}&=R(\zeta ,\sigma _A^2,\sigma _B^2,\eta ,\upsilon _{\text {el}},T_A,T_B,\Xi ), \end{aligned}$$

which is calculated starting from the CM in Eq. (17) as in Ref.^21^. Note that $\zeta$ is the reconciliation parameter defined later in Eq. (70). This parameter accounts for the proportion of mutual information given to Eve during the public channel communication of the parties performing a non-ideal reconciliation process.

### Parameter estimation

Here we follow the PE proposed in Ref.^33^. An alternative way is described in^27^, Appendix II.B based on extra simplifying assumptions. In particular, based on *m* samples $[Q_A]_i$, $[Q_B]_i$, $[Q_R]_i$, for $i=1,\dots , m$, the parties calculate the maximum likelihood estimators (MLEs) of the covariances $\text {Cov}( Q_A, Q_R)=\langle Q_A Q _R\rangle =-\tau _A \sigma _A^2$ and $\text {Cov}( Q_B, Q_R)=\langle Q_B Q _R\rangle =\tau _ B\sigma _B^2$. These estimators are given by

44 $$\begin{aligned} \widehat{C}_{Q_AQ_R}&=\frac{1}{m}\sum _{i=1}^m [Q_A]_i[Q_R]_i , \end{aligned}$$

45 $$\begin{aligned} \widehat{C}_{Q_BQ_R}&=\frac{1}{m}\sum _{i=1}^m [Q_B]_i[Q_R]_i , \end{aligned}$$

46 $$\begin{aligned} \widehat{C}_{P_AP_R}&=\frac{1}{m}\sum _{i=1}^m [P_A]_i[P_R]_i, \end{aligned}$$

47 $$\begin{aligned} \widehat{C}_{P_BP_R}&=\frac{1}{m}\sum _{i=1}^m [P_B]_i[P_R]_i. \end{aligned}$$

From these, they define estimators for $T_A$ and $T_B$, i.e.,

48 $$\begin{aligned} \widehat{T}_{A}&=\frac{2 }{\eta _\text {eff}(\sigma _A^2)^2}\text {min}\{|\widehat{C}_{Q_AQ_R}|^2,|\widehat{C}_{P_AP_R}|^2\}, \end{aligned}$$

49 $$\begin{aligned} \widehat{T}_{B}&=\frac{2 }{\eta _\text {eff}(\sigma _B^2)^2}\text {min}\{|\widehat{C}_{Q_BQ_R}|^2,|\widehat{C}_{P_BP_R}|^2\}. \end{aligned}$$

Then they define an estimator for $\sigma ^2_{z}$. This estimator is given by

50 $$\begin{aligned} \widehat{\sigma }_{z}^2=&\max \left\{ \frac{1}{m}\sum _{i=1}^m \left( [Q_R]_i+\widehat{\tau }_A[Q_A]_i-\widehat{\tau }_B[Q_B]_i\right) ^2\right. ,\nonumber \\&\left. \frac{1}{m}\sum _{i=1}^m \left( [P_R]_i-\widehat{\tau }_A[P_A]_i-\widehat{\tau }_B[P_B]_i\right) ^2\right\} , \end{aligned}$$

with

51 $$\begin{aligned} \widehat{\tau }_A&=\sqrt{\eta _\text {eff}\widehat{T}_{A}/2}, \end{aligned}$$

52 $$\begin{aligned} \widehat{\tau }_B&=\sqrt{\eta _\text {eff}\widehat{T}_{B}/2}, \end{aligned}$$

and obtain the associated variances^27^, Appendix II.A

53 $$\begin{aligned} \sigma ^2_{\widehat{T}_{A}}&\simeq \frac{4 \widehat{T}_A }{m}\left[ \widehat{T}_A+\frac{\widehat{T}_B}{2}\frac{\sigma ^2_B}{\sigma _A^2}\right] \left( 2 +\frac{2\widehat{\sigma }_z^2/\eta _\text {eff}}{\widehat{T}_A \sigma _A^2+\frac{\widehat{T}_B}{2}\sigma _B^2} \right) , \end{aligned}$$

54 $$\begin{aligned} \sigma ^2_{\widehat{T}_{B}}&\simeq \frac{4 \widehat{T}_B }{m}\left[ \widehat{T}_B+\frac{\widehat{T}_A}{2}\frac{\sigma ^2_A}{\sigma _B^2}\right] \left( 2 +\frac{2\widehat{\sigma }_z^2/\eta _\text {eff}}{\widehat{T}_B \sigma _B^2+\frac{\widehat{T}_A}{2}\sigma _A^2} \right) , \end{aligned}$$

55 $$\begin{aligned} V_{z}&\simeq \frac{2 (\widehat{\sigma }_{z}^2)^2}{m}. \end{aligned}$$

Based on $\widehat{\sigma }_z^2$ they find an estimator for $\Xi$ given by

56 $$\begin{aligned} \widehat{\Xi }=\widehat{\sigma }_z^2-v_\text {el}-1, \end{aligned}$$

with variance equal to

57 $$\begin{aligned} \sigma ^2_{\widehat{\Xi }}:=V_{z}. \end{aligned}$$

Finally, worst-case scenario values can be derived given the PE error $\epsilon _\text {PE}$. These values are

58 $$\begin{aligned} \widetilde{T}_A&=\widehat{T}_A-w\sigma _{\widehat{T}_A}, \end{aligned}$$

59 $$\begin{aligned} \widetilde{T}_B&=\widehat{T}_B-w\sigma _{\widehat{T}_B}, \end{aligned}$$

60 $$\begin{aligned} \widetilde{\Xi }&= \widehat{\Xi }+w\sigma _{\widehat{\Xi }}, \end{aligned}$$

where

61 $$\begin{aligned} w=\sqrt{2}\text {erf}^{-1}(1-\epsilon _\text {PE}). \end{aligned}$$

Using the previous values, the parties can compute a secret key rate with an overestimated Holevo information

62 $$\begin{aligned} R_m:&=\zeta I(\textbf{x}:\textbf{y})|_{\widehat{T}_A,\widehat{T}_B,\widehat{\Xi }} -\chi (E:\textbf{y}|\varvec{\gamma })|_{\widetilde{T}_A,\widetilde{T}_B,\widetilde{\Xi }}. \end{aligned}$$

Note here that $m=N-n$ where *N* is the number of signals sent through the channel and *n* is the number of signals devoted to secret key extraction for each block. In a practical situation, where the transmission can be assumed stable over a large number of blocks $n_{\text {bks}}$, one can use *m* signals on average from each block in order to estimate the channel parameters. Thus the parties sacrifice $M=m n_\text {bks}$ for PE and the corresponding rate is given by

63 $$\begin{aligned} R_m \rightarrow R_M:&=\left[ \zeta I(\textbf{x}:\textbf{y})|_{\widehat{T}_A,\widehat{T}_B,\widehat{\Xi }}\right. \nonumber \\&\quad \left. -\chi (E:\textbf{y}|\varvec{\gamma })|_{\widetilde{T}_A,\widetilde{T}_B,\widetilde{\Xi }} \right] _{m=M}. \end{aligned}$$

The mutual information and the correlation between the two variables $\textbf{x}$ and $\textbf{y}$ are connected as follows^38^, Eq. (8.56) (see also^27^, Eq. (2)):

64 $$\begin{aligned} I(\textbf{x}:\textbf{y})= \log _2 \left[ 1+\text {SNR}\right] =\log _2 \left[ (1-\rho _{\textbf{xy}}^2)^{-1}\right] . \end{aligned}$$

One may derive the estimator for the correlation between the variables by replacing with the MLEs of the transmissivities and noise into the mutual information, namely,

65 $$\begin{aligned} \widehat{\rho }_{\textbf{xy}}= \sqrt{1-2^{-I(\textbf{x}:\textbf{y})|_{\widehat{T}_A,\widehat{T}_B,\widehat{\Xi }}}}, \end{aligned}$$

which helps in the calculation of the *a priori* probabilities for the initialization step of the decoding sum-product algorithm of the error correction step^31,32^.

### Data reconciliation

The parties apply the transformations of Eqs. (29)–(32) based on the quantities in Eqs. (34)–(37) calculated via the MLEs of the previous section. Bob and Alice combine their data from the *Q* and *P* quadratures into one variable. In particular, Alice and Bob apply the following mapping to their data:

66 $$\begin{aligned} {[}x]_{2i-1}&=[Q_x]_i, \end{aligned}$$

67 $$\begin{aligned} {[}x]_{2i}&=[P_x]_i, \end{aligned}$$

68 $$\begin{aligned} {[}y]_{2i-1}&=[Q_y]_i, \end{aligned}$$

69 $$\begin{aligned} {[}y]_{2i}&=[P_y]_i, \end{aligned}$$

in order to obtain 2*n* samples from each block. Afterwards, the parties apply the EC procedure using non-binary LDPC codes following Ref.^32^, Sect. III.B (for extra details see also Ref.^31^). More specifically, they define the worst-case estimator (up to an error probability $\epsilon _\text {ent}$) for the reconciliation parameter $\zeta$ appearing in Eq. (43) which is given by

70 $$\begin{aligned} \hat{\zeta }=2\frac{\widehat{H}(\mathsf{l})+R_\text {code} q-p- \delta _\text {ent}}{I(\textbf{x}:\textbf{y})|_{\widehat{T}_A,\widehat{T}_B,\widehat{\Xi }}}, \end{aligned}$$

where $2(\widehat{H}(\mathsf{l})-\delta _\text {ent})$ is the worst-case scenario entropy of the raw-key string described by $\textsf{l}$, the normalized and discretized version of *y*. In particular, $2\widehat{H}(\mathsf{l})$ is the estimator of the previous entropy, $-R_\text {code}q+p$ is the maximum data exchanged for reconciliation per channel use when one uses a non-binary LDPC code with the rate $R_\text {code}$ associated with the Galois filed $\mathcal {G}(2^q)$ and discretization of *p* bits. We take into consideration here that Bob applies the LDPC encoding only to the *q* bits of $\mathsf{l}$ while the rest $p-q$ bits are entirely sent through the public channel. $I(\textbf{x}:\textbf{y})|_{\widehat{T}_A,\widehat{T}_B,\widehat{\Xi }}$ is the ideal mutual information between the parties according to the data (i.e., after parameter estimation) which appears in Eq. (63). In fact by replacing $\hat{\zeta }$ in the previous equation, one obtains the practical key rate

71 $$\begin{aligned} R_M^\text {EC}=2[\widehat{H}(\mathsf{l})+R_\text {code} q-p-\delta _\text {ent}]-\chi (E :\textbf{y}|\varvec{\gamma })|_{\widetilde{T}_A,\widetilde{T}_B,\widetilde{\Xi }}. \end{aligned}$$

The parties started with two different sequences of $n_\text {bks}$ blocks each with 2*N* initial samples and, in the process (after PE and EC), these are reduced to two indistinguishable binary sequences (with probability $1-\epsilon _\text {EC}$) that consist of $p_\text {EC}n_\text {bks}$ blocks each carrying 2*np* bits:

72 $$\begin{aligned} \mathsf{l}_\text {bin}:=\overline{\mathsf{l}}_\text {bin}^n\underline{\mathsf{l}}_\text {bin}^n\simeq \tilde{\mathsf{l}}_\text {bin}:=\widehat{\mathsf{l}}_\text {bin}^n\underline{\mathsf{l}}_\text {bin}^n. \end{aligned}$$

Note that $\overline{\mathsf{l}}_\text {bin}^n$ corresponds to the part of the original variable $\mathsf{l}$ in binary form that has been sent through the public channel using the LDPC encoding, $\underline{\mathsf{l}}_\text {bin}^n$ is the part in binary form that has been sent unchanged through the public channel, and $\widehat{\mathsf{l}}_\text {bin}^n$ is the binary form of the successfully decoded and verified part with probability $p_\text {EC}$. The parties need to apply on these sequences the appropriate amount of compression during the PA step so that the previous raw-data strings become a secret key. This is determined by the composable key rate calculated below. Concatenating appropriately the previous parts, the parties end up with the raw data sequences $\mathsf{l}_\text {bin}$ for Bob and $\tilde{\mathsf{l}}_\text {bin}$ for Alice in binary form.

### Composable security

We adopt the composable framework security analysis presented in Ref.^17^, Appendix G to the requirements of the CV-MDI-QKD protocol. More specifically, the secret key is characterized by certain properties stemming from certain post-processing procedures, and there is an overall probability $\epsilon$ that the key fails to possess at least one of these properties.

According to the previous analysis, one may write for the length of the secret key^17^, Eq. (G12):

73 $$\begin{aligned} s_n&\ge n H(l|E\varvec{\gamma })_{\rho }-\sqrt{n}\Delta _\text {AEP}(p_\text {EC}\epsilon _\text {s}^2/3,2^{2p})\nonumber \\ {}&\quad +\log _2\left[ p_\text {EC}(1-\epsilon^2_\text{s}/3)\right] +2\log _2\sqrt{2}\epsilon _\text {h}-\text {leak}_\text {EC}, \end{aligned}$$

where *l* is defined according to the bidirectional mapping

74 $$\begin{aligned} l=\mathsf{l}_Q2^p+\mathsf{l}_P, \end{aligned}$$

where $\mathsf{l}_{Q}$($\mathsf{l}_{P}$) is the $\textsf{l}$ instance corresponding to the *q*(*p*)-quadrature. Note that here we have used a virtual concatenation assumption (see Appendix A of ^32^) to pass from a description based on the single-quadrature variable $\textsf{l}$ (normalized and discetized) to one based on the vectorial variable *l*. One may also observe that, in this case, Eve’s system is described by the group of modes *E* plus the classical variable $\varvec{\gamma}$. In particular, $H(l|{E\varvec{\gamma }})$ is the conditional von Neumann entropy of the variable *l* conditioned on *E* and $\varvec{\gamma}$, and^39^, Eq. (61)

75 $$\begin{aligned} \Delta _\text {AEP}(\epsilon _\text {s},|\mathcal {L}|)=4\log _2(\sqrt{|\mathcal {L}|}+2)\sqrt{\log _2(2/\epsilon _s^2)}, \end{aligned}$$

with $|\mathcal {L}|$ being the cardinality of the discretized variable *l*, which in our case is $2^{2p}$. Note that, for the conditional mutual information, we have ^37^, Def. 7.4.1

76 $$\begin{aligned} I(l:E|\varvec{\gamma })_{\rho _l}=H(l|\varvec{\gamma })-H(l|E \varvec{\gamma })_{\rho _l}. \end{aligned}$$

In particular, this mutual information is between a classical variable *l* and a quantum system *E* (conditioned on another classical variable $\varvec{\gamma }$). This is therefore the Holevo information $\chi (E:l|\varvec{\gamma })$, i.e., an upper bound for the accessible information on *l* given that Eve possesses *E* (and knows the variable $\varvec{\gamma }$). Therefore, by reversing Eq. (76), one may write

77 $$\begin{aligned} H(l|{E}\varvec{\gamma })_{\rho _l}=H(l|\varvec{\gamma })-\chi (E:l|\varvec{\gamma })_{\rho _l}, \end{aligned}$$

where $H(l|\varvec{\gamma })=H(l)$ (see Eq. 33) is the Shannon entropy of *l*. In more detail, using the data processing inequality, we manipulate Eve’s Holevo bound as follows

78 $$\begin{aligned} \chi (E:l|\varvec{\gamma })_{\rho _l}\le \chi (E:Q_y,P_y|\varvec{\gamma })=\chi (E:\textbf{y}|\varvec{\gamma }). \end{aligned}$$

Therefore we have

79 $$\begin{aligned} H(l|E\varvec{\gamma })_{\rho _l} \ge H(l)-\chi (E:\textbf{y}|\varvec{\gamma }). \end{aligned}$$

We may replace Eq. (79) in Eq. (73) and then set

80 $$\begin{aligned} \zeta I(\textbf{x}:\textbf{y})=H(l)-n^{-1}\text {leak}_\text {EC}. \end{aligned}$$

In this way, we derive

81 $$\begin{aligned} s_n&\ge n R_\text {asy}-\sqrt{n}\Delta _\text {AEP}(p_\text {EC}\epsilon _\text {s}^2/3,2^{2p})\nonumber \\ {}& \quad + \log _2[p_\text {EC}(1-\epsilon ^2_\text {s}/3)]+2\log _2\sqrt{2}\epsilon _\text {h}, \end{aligned}$$

where we include the asymptotic secret key rate of Eq. (43). One may replace $R_\text {asy}$ with $R_{M}^{\textrm{EC}}$ of Eq. (71) into Eq. (81) to obtain (see also^17,32,39^)

82 $$\begin{aligned} R=\frac{np_{\text {EC}}}{N}\tilde{R},~\tilde{R}:=\left( R_{M}^{\textrm{EC}}-\frac{\Delta _{\text {AEP}}}{\sqrt{n}}+\frac{\Theta }{n}\right) , \end{aligned}$$

with composable terms

83 $$\begin{aligned} \Delta _{\text {AEP}}&:=4\log _{2}\left( 2^{p} + 2\right) \sqrt{\log _{2}\left( \frac{18}{p_{\text {EC}}^{2}\epsilon _{\text {s}}^{4}}\right) }, \end{aligned}$$

84 $$\begin{aligned} \Theta&:=\log _{2}[p_{\text {EC}}(1-\epsilon _{\text {s}}^{2}/3)]+2\log _{2}\sqrt{2}\epsilon _{\text {h}}. \end{aligned}$$

The overall security parameter is equal to

85 $$\begin{aligned} \epsilon = \epsilon _\text {cor}+\epsilon _\text {h}+\epsilon _\text {s}+p_\text {EC}(3\epsilon _\text {PE}+\epsilon _\text {ent}), \end{aligned}$$

where we note that the factor 3 is due to the fact the $\epsilon _\text {PE}$ is defined per parameter.

One may also derive an approximate key rate, which is not based on the data postprocessing

86 $$\begin{aligned} R_\text {theo}=\frac{np_{\text {EC}}}{N}R^\star ,~R^\star :=\bar{R}_{M}-\frac{\Delta _{\text {AEP}}}{\sqrt{n}}+\frac{\Theta }{n}, \end{aligned}$$

where $\bar{R}_{M}$ is the rate in Eq. (63) but where the estimators are approximated using the initial values of the simulation (see, e.g., the steps in Sects. III.B.1 and III.B.2 in Ref.^31^). In fact, one may define $\bar{R}_{M}$ from Eq. (63) but where the following substitutions have been made:

87 $$\begin{aligned} \widehat{T}_A&\rightarrow \mathbb {E}(\widehat{T}_A)\simeq T_A+\mathcal {O}(1/M), \end{aligned}$$

88 $$\begin{aligned} \widehat{T}_B&\rightarrow \mathbb {E}(\widehat{T}_B)\simeq T_B+\mathcal {O}(1/M), \end{aligned}$$

89 $$\begin{aligned} \widehat{\Xi }&\rightarrow \mathbb {E}(\widehat{\Xi })\simeq \Xi , \end{aligned}$$

and

90 $$\begin{aligned} \widetilde{T}_A&\rightarrow T_A-w\sigma _{T_A}, \end{aligned}$$

91 $$\begin{aligned} \widetilde{T}_B&\rightarrow T_B-w\sigma _{T_B}, \end{aligned}$$

92 $$\begin{aligned} \widetilde{\Xi }&\rightarrow \Xi +w\sigma _{\Xi }, \end{aligned}$$

where $\sigma _{T_A}$, $\sigma _{T_B}$, and $\sigma _{\Xi }$ have been calculated through Eqs. (53),  (54), and (57), respectively, after replacing $T_A$, $T_B$, and $\sigma _z^2$ in those formulas. Note that the rates presented in this section do not rely on the conjecture mentioned in^27^, Appendix VI.

### Privacy amplification

Now the parties are ready to apply the appropriate amount of compression indicated by Eq. (82) on their binary strings in Eq. (72) to create a secret key through the PA step. To achieve this, they compress them via universal hashing. More specifically, they apply a modified Toeplitz matrix $\textbf{G}(\textbf{I}_r|\textbf{T}_{r,2np-r})$ to their sequences in order to extract the secret key^40^

93 $$\begin{aligned} K=\textbf{G} S= \textbf{I}_r \mathsf{l}_\text {bin}^{r} \oplus \textbf{T}_{r,2np-r} \mathsf{l}_\text {bin}^{2np-r}, \end{aligned}$$

where $r=2np\tilde{R}$, the Toeplitz matrix $\textbf{T}_{r,2np-r}$ is of $r\times 2np-r$ dimensions and $\textsf{I}_r$ is the $r \times r$ identity matrix, with $\textsf{l}_\text {bin}^{r}$ we denote the first *r* bits of the raw key string and with $\textsf{l}_\text {bin}^{2np-r}$ the rest.

## Simulation and results

In our simulations, the attack is handled by initially defining values for the excess noise of Alice’s ($\xi _{A}$) and Bob’s ($\xi _{B}$) channels. These values, along with the transmissivity of each channel, constitute the thermal noise $\omega$ for each channel respectively as follows:

94 $$\begin{aligned} \omega _{A}&= \dfrac{T_{A}\xi _{A}}{1 - T_{A}} + 1, \end{aligned}$$

95 $$\begin{aligned} \omega _{B}&= \dfrac{T_{B}\xi _{B}}{1 - T_{B}} + 1. \end{aligned}$$

Using Alice’s thermal noise value, we can estimate the correlation parameter *g* from Eq. (11). We now have all components to find the excess noise variance $\Xi$, which is shown in Eq. (10). Finally, the noise variance $\sigma ^2_{z}$ is calculated through Eq. (9).

The parameters used to execute the simulations are listed in Table 2. To begin with, the symmetric version of the protocol is examined, which means that the signal variance and the channel parameters will be the same between Alice and Bob, i.e. $\mu _{A}=\mu _{B}$, $T_{A}=T_{B}$ and $\xi _{A}=\xi _{B}$.

To find a signal variance range, for which the composable rate *R* becomes positive, the asymptotic rate $R_\text {asy}$ was maximized using a modulation variance optimization function. Table 1 shows that a positive *R* can be achieved, when $45\le \mu _{A}, \mu _{B} \le 50$. Under these conditions, the SNR spans from approximately 10 to 11.89. As presented in the Table, the choice of the reconciliation efficiency is important, when trying to maximize the value of *R*. It is important to note that neither the asymptotic nor the composable rate will further grow, as the signal variances increase. This means that, at some point, the rates will saturate and eventually become negative again.

**Table 1:** Composable secret key rate *R* (bits/use) versus Alice’s and Bob’s signal variances $\mu _{A}$ and $\mu _{B}$. The rightmost column displays the average value for *R*, which is obtained after 5 simulations. Here we use $N=5 \times 10^{5}$ and $n_\text {bks}=100$. All simulations have achieved $p_\text {EC} \ge 0.95$. Parameters not listed here are taken as in Table 2.

$\mu _{A}$, $\mu _{B}$	$\zeta$ (%)	$R_\text {code}$	$\text {SNR}$	*R*
45	$90$	0.833	10.019	0.00452259
46	$92.15$	0.846	10.252	0.06346475
47	$91.35$	0.846	10.485	0.04397952
48	$90.62$	0.846	10.718	0.01369927
49	$92.35$	0.857	10.951	0.06547397
50	$91.64$	0.857	11.189	0.04992091

**Figure 2:**
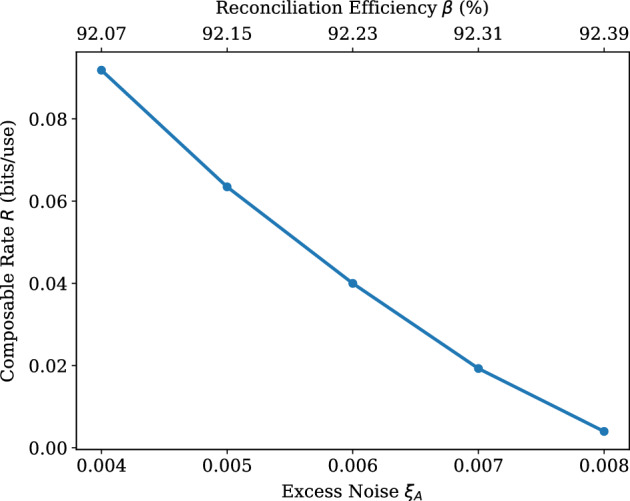
Composable secret key rate *R* (bits/use) versus Alice’s and Bob’s excess noise values $\xi =\xi _{A}=\xi _{B}$. Every point represents the average value of *R*, which is obtained after 5 simulations. Here we use $N=5 \times 10^{5}$ and $n_\text {bks}=100$. All simulations have achieved $p_\text {EC} \ge 0.95$. The signal variances used by Alice and Bob are constant and equal ($\mu _{A}=\mu _{B}=46$). The values of the reconciliation efficiency $\zeta$ are shown on the top axis. Other parameters are taken as in Table 2.

Knowing the variables, for which the composable rate becomes positive, we can now identify what is the maximum tolerable excess noise in the system. For this purpose, $\mu _{A} = \mu _{B} = 46$ is chosen, in order to produce a high rate (and therefore tolerate more excess noise), while performing a faster EC procedure (when compared to that for $\mu _{A} = \mu _{B} = 49$). Therefore, in Fig. 2, the symmetric case of the protocol is considered again, with $\mu _{A}=\mu _{B}=46$ and with the excess noise being variable. As observed from the plot, $\xi$ can take values up to 0.008, before the protocol is deemed unsafe for key distribution.

**Table 2:** The input parameters for the simulations.

Parameter	Value (Table 1)	Value (Fig. 2)	Value (Fig. 3)	Value (Fig. 4)
$T_{A}$	0.98	0.98	Variable	0.96
$T_{B}$	0.98	0.98	0.985	0.985
$\xi _{A}$	0.005	Variable	0.006	Variable
$\xi _{B}$	0.005	Variable	0.004	0.004
$\eta$	0.98	0.98	0.98	0.98
$\upsilon _{\text {el}}$	0.01	0.01	0.01	0.01
$n_{\text {bks}}$	100	100	100	100
*N*	$5 \times 10^{5}$	$5 \times 10^{5}$	$5.88 \times 10^{5}$	$5.88 \times 10^{5}$
*M*	$0.1n_{\text {bks}}N$	$0.1n_{\text {bks}}N$	$0.15n_{\text {bks}}N$	$0.15n_{\text {bks}}N$
*p*	6	6	7	7
*q*	4	4	4	4
$\alpha$	7	7	7	7
$\text {iter}_{\text {max}}$	200	200	100	100
$\epsilon _{\text{PE}, \text{s}, \text{h}, \text{corr}}$	$2^{-32}$	$2^{-32}$	$2^{-32}$	$2^{-32}$
$\mu _{A}$	Variable	46	60	60
$\mu _{B}$	Variable	46	50	50

**Figure 3:**
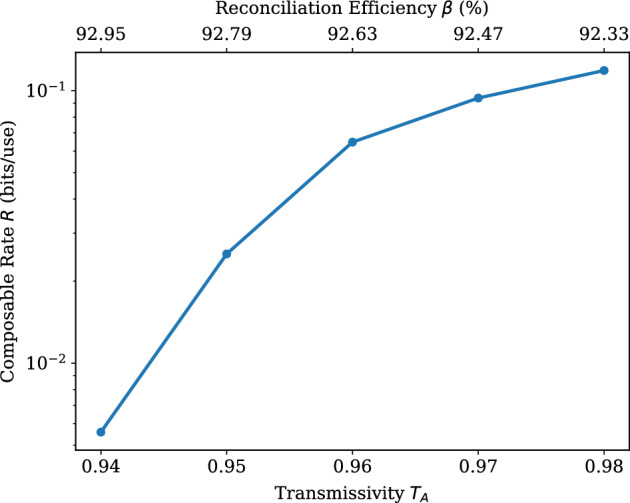
Composable secret key rate *R* (bits/use) versus Alice’s transmissivity $T_{A}$. Every point represents the average value of *R*, which is obtained after 5 simulations. Here we use $N=5.88 \times 10^{5}$ and $n_\text {bks}=100$. All simulations have achieved $p_\text {EC} \ge 0.95$. The signal variances used by Alice and Bob are constant ($\mu _{A}=60$, $\mu _{B}=50$). The values of the reconciliation efficiency $\zeta$ are shown on the top axis. Other parameters are taken as in Table 2.

**Figure 4:**
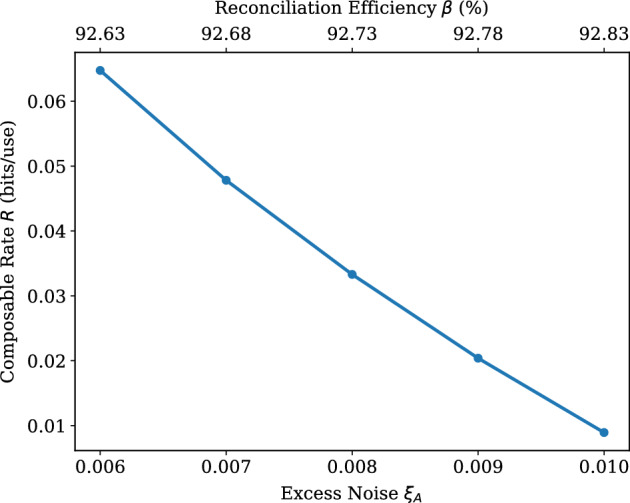
Composable secret key rate (bits/use) versus Alice’s excess noise value $\xi _{A}$. Every point represents the average value of *R*, which is obtained after 5 simulations. Here we use $N=5.88 \times 10^{5}$ and $n_\text {bks}=100$. All simulations have achieved $p_\text {EC} \ge 0.95$. The signal variances used by Alice and Bob are constant ($\mu _{A}=60$, $\mu _{B}=50$). The values of the reconciliation efficiency $\zeta$ are shown on the top axis. Other parameters are taken as in Table 2.

Next, we investigate the asymmetric version of the protocol, where the channel parameters, as well as the signal variances, are different between Alice and Bob. Here, two cases are examined: Fig. 3 shows the behaviour of Alice’s transmissivity against the composable key rate and Fig. 4 displays the maximum tolerable values for Alice’s excess noise. Regarding the former case, it is possible for Alice’s channel to reach transmissivity values of about $T_{A} = 0.94$, which translates to a fiber length of 1.34 km. The latter case shows that it is feasible to achieve a positive *R* under relatively high values for the excess noise, which can be extended to $\xi _{A}=0.01$. To ensure a positive composable rate is positive under harsher noise settings, it is possible to employ a larger LDPC matrix with a block length very close to the order of $10^6$ (The use of non-binary LDPC codes allows for block sizes under $10^6$. A fair comparison with existing research (using binary LDPC codes) would be to multiply the current block sizes with the Galois field component q. Note that the stable channel assumption (see Eq. 63) and the use of high SNR in our study contribute, as well, to obtaining rates with these block size values.) and $R_\text {code}=0.875$ for the task. Because of the finite-size effect, a larger LDPC block size leads to higher values for the reconciliation efficiency, when all other values remain the same.

## Conclusion

In this study, we give a rigorous proof for the composable security of the Gaussian-modulated CV-MDI protocol and we calculate its rate. Depending on this rate, the appropriate amount of compression is applied, in order to extract a secret key. We simulate the quantum communication step and we apply all the classical postprocessing steps on the generated data. All of these procedures are performed by means of an associated Python library. This library allows us to calibrate and optimize all the relevant parameters with direct benefits for experimental implementations.

## Supplementary Information

Supplementary Information.

## Data Availability

The datasets and the Python library used and/or analyzed during the current study are available from the corresponding author upon reasonable request.
