# Supplementary material for: Composable security of CV-MDI-QKD with secret key rate and data processing
Source: Sci Rep. 2023 Jul 19;13:11636. doi: 10.1038/s41598-023-37699-5 (PMC10356849; doi:10.1038/s41598-023-37699-5)
Supplement: Supplementary file 1 — Supplementary Information. [file 41598_2023_37699_MOESM1_ESM.pdf]

Supplementary material:
Composable security of CV-MDI-QKD with Secret key rate and data processing

Panagiotis Papanastasiou,* Alexander G. Mountogiannakis, and Stefano Pirandola
Department of Computer Science, University of York, York YO10 5GH, United Kingdom

I. EQUIVALENT MUTUAL INFORMATION

The CM of $\mathbf{x}$ and $\mathbf{y}$ is given by

$$\Sigma_{\mathbf{xy}} = \begin{pmatrix} \sigma_x^2\mathbf{I} & \sigma_{xy}\mathbf{Z} \\ \sigma_{xy}\mathbf{Z} & \sigma_y^2\mathbf{I} \end{pmatrix} = \begin{pmatrix} \left(\sigma_A^2 - \frac{\tau_A^2(\sigma_A^2)^2}{\tau_A^2\sigma_A^2+\tau_B^2\sigma_B^2+\sigma_z^2}\right)\mathbf{I} & \frac{\tau_A\tau_B\sigma_A^2\sigma_B^2}{\tau_A^2\sigma_A^2+\tau_B^2\sigma_B^2+\sigma_z^2}\mathbf{Z} \\ \frac{\tau_A\tau_B\sigma_A^2\sigma_B^2}{\tau_A^2\sigma_A^2+\tau_B^2\sigma_B^2+\sigma_z^2}\mathbf{Z} & \left(\sigma_B^2 - \frac{\tau_B^2(\sigma_B^2)^2}{\tau_A^2\sigma_A^2+\tau_B^2\sigma_B^2+\sigma_z^2}\right)\mathbf{I} \end{pmatrix}. \tag{1}$$

One may apply the formula for the mutual information [1] for bivariate normal distributions, taking into consideration that the quadratures are independent and [2, Eq. (64)], so that

$$I(\mathbf{x}:\mathbf{y}) = \left(\frac{1}{2}\log_2\left[1-(\rho^Q_{\mathbf{xy}})^2\right]^{-1}\right) + \left(\frac{1}{2}\log_2\left[1-(\rho^P_{\mathbf{xy}})^2\right]^{-1}\right) \tag{2}$$

$$= \log_2\left[1-\rho^2_{\mathbf{xy}}\right]^{-1}, \tag{3}$$

with

$$\rho_{\mathbf{xy}} = \rho^Q_{\mathbf{xy}} = -\rho^P_{\mathbf{xy}} = \frac{\sigma_{xy}}{\sqrt{\sigma_x^2}\sqrt{\sigma_y^2}} = \tau_A\tau_B\sqrt{\frac{\sigma_A^2\sigma_B^2}{(\tau_A^2\sigma_A^2+\sigma_z^2)(\tau_B^2\sigma_B^2+\sigma_z^2)}}. \tag{4}$$

We can then verify that it is equivalent to [2, Eq. (23)].

II. CHANNEL PARAMETER ESTIMATION

A. Calculation of MLEs variances

From [2, Eq. (44)], we have (see also the method in Ref. [3])

$$\mathrm{Var}(\widehat{C}_{Q_AQ_R}) = \frac{1}{m^2}\sum_{i=1}^m \mathrm{Var}([Q_A]_i[Q_R]_i)$$
$$= \frac{1}{m}\left[\tau_B^2\langle Q_B^2Q_A^2\rangle + 2\tau_A^2\langle Q_A^2\rangle^2 + \langle Q_z^2Q_A^2\rangle\right], \tag{5}$$

where, after replacing from [2, Eqs. (5, 6)], we obtain

$$\mathrm{Var}(\widehat{C}_{Q_AQ_R}) = \frac{\frac{(\sigma_A^2)^2}{m}\left[\tau_A^2 + \frac{\tau_B^2}{2}\frac{\sigma_B^2}{\sigma_A^2}\right]}{\left(2+\frac{\sigma_z^2}{\tau_A^2\sigma_A^2+\frac{\tau_B^2}{2}\sigma_B^2}\right)^{-1}} := V_{Q_AQ_R}. \tag{6}$$

Similar calculations hold for [2, Eqs. (45, 46, 47)]. In case that $|\widehat{C}_{Q_AQ_R}| < |\widehat{C}_{P_AP_R}|$ (see [2, Eq. (48)]), we have

$$\widehat{T}_A = \frac{2V_{Q_AQ_R}}{\eta_{\text{eff}}(\sigma_A^2)^2}\left(\frac{\widehat{C}_{Q_AQ_R}}{\sqrt{V_{Q_AQ_R}}}\right)^2. \tag{7}$$

The variable $\left(\frac{\widehat{C}_{Q_AQ_R}}{\sqrt{V_{Q_AQ_R}}}\right)^2$ is a chi-squared distributed variable with variance

$$2\left(1+2\left(\frac{C_{Q_AQ_R}}{\sqrt{V_{Q_AQ_R}}}\right)^2\right), \tag{8}$$

such that

$$\mathrm{Var}(\widehat{T}_A) = \frac{16V_{Q_AQ_R}C^2_{Q_AQ_R}}{\eta^2_{\text{eff}}(\sigma_A^2)^4} + \mathcal{O}(1/m^2). \tag{9}$$

Otherwise, $\widehat{T}_A$ is expressed by P_A and P_R and we obtain a similar relation for $\mathrm{Var}(\widehat{T}_A)$. We obtain [2, Eq. (53)] after making all the appropriate replacements. With similar steps, we write [2, Eq. (54)] for $\widehat{T}_B$.

Based on [2, Eq. (50)] and given that

$$\frac{1}{m}\sum_{i=1}^m\left([q_R]_i + \widehat{\tau}_A[q_A]_i - \widehat{\tau}_B[q_B]_i\right)^2$$
$$> \frac{1}{m}\sum_{i=1}^m\left([p_R]_i - \widehat{\tau}_A[p_A]_i - \widehat{\tau}_B[p_B]_i\right)^2, \tag{10}$$

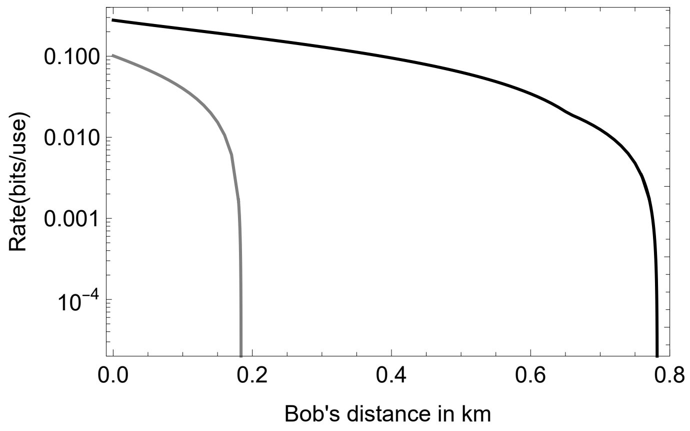

FIG. 1: We plot the composable secret key rate $R_{\rm theo}$ (black solid line) from [2, Eq. (86)] for $\beta = 0.9188$ and $p_{\rm EC} = 0.95$. We have assumed $p = 6$, and $N = 5.175 \times 10^5$ while the rest parameters have been taken from Table II for Fig. 3. For the sake of comparison, we plot the rate from Ref. [5] for the same parameters (gray line).

we have

$$\begin{aligned}\mathrm{Var}(\widehat{\sigma}_z^2) &= \mathrm{Var}\left[\frac{1}{m}\sum_{i=1}^{m}\left([q_R]_i + \widehat{\tau}_A[q_A]_i - \widehat{\tau}_B[q_B]_i\right)^2\right] \\ &\simeq \mathrm{Var}\left[\frac{\sigma_z^2}{m}\sum_{i=1}^{m}\left(\frac{[q_R]_i + \tau_A[q_A]_i - \tau_B[q_B]_i}{\sqrt{\sigma_z^2}}\right)^2\right] \\ &= \frac{(\sigma_z^2)^2}{m^2}2m = \frac{2}{m}(\sigma_z^2)^2. \end{aligned} \quad (11)$$

Above we assumed that the estimators $\widehat{\tau}_A$ and $\widehat{\tau}_B$ have negligible variances and can be replaced with their true values, so that any uncertainty for σ_z^2 stems purely from the data. We then observe that the sum in the same equation can be considered to be a chi-squared distributed variable with variance equal to $2m$. Finally, we obtain [2, Eq. (56)], by solving [2, Eq. (9)] with respect to Ξ and taking its variance. We then use Eq. (11) with σ_z^2 replaced by its estimator. Similar calculations hold for the other direction of the inequality in Eq. (10).

B. PE with extra simplifying assumptions

Alice and Bob declare m instances $\{q_{Ai}\}$, $\{p_{Ai}\}$ and $\{q_{Bi}\}$, $\{p_{Bi}\}$ for $i = 1, \ldots, m$ of their local variables. Using the relative relay output instances $\{q_{Ri}\}$, $\{p_{Ri}\}$, they estimate τ_A, τ_B, σ_z and σ_z'. In particular, according to the multiple linear regression model [4], one obtains the following MLEs:

$$\widehat{\tau}_{A_q} = -\frac{\sum_{i=1}^m q_{Ai}q_{Ri}}{\sum_{i=1}^m q_{Ai}^2}, \quad \widehat{\tau}_{A_p} = \frac{\sum_{i=1}^m p_{Ai}p_{Ri}}{\sum_{i=1}^m p_{Ai}^2} \quad (12)$$

$$\widehat{\tau}_{B_q} = \frac{\sum_{i=1}^m q_{Bi}q_{Ri}}{\sum_{i=1}^m q_{Bi}^2}, \quad \widehat{\tau}_{B_p} = \frac{\sum_{i=1}^m p_{Bi}p_{Ri}}{\sum_{i=1}^m p_{Bi}^2} \quad (13)$$

$$\widehat{\sigma}_z^2 = \frac{1}{m}\sum_{i=1}^{m}(q_{Ri} - \widehat{\tau_B}q_{Bi} - \widehat{\tau_A}q_{Ai})^2 \quad (14)$$

$$\widehat{\sigma}_{z'}^2 = \frac{1}{m}\sum_{i=1}^{m}(p_{Ri} - \widehat{\tau}_B p_{Bi} - \widehat{\tau}_A p_{Ai})^2. \quad (15)$$

This is true for the MLEs for τ_A and τ_B, because Alice modulates her mode independently from Bob. Then, from the theory of linear multiple regression, this is true for τ_{Aq} and τ_{Bq} and the MLE for σ_z^2. The same reasoning holds for $\widehat{\tau_{Ap}}$, $\widehat{\tau_{Bp}}$ and $\sigma_{z'}^2$. The previous MLEs are distributed according to

$$\widehat{\tau}_{A_q} \sim \mathcal{N}(\tau_A, \frac{\widehat{\sigma_z^2}}{m\sigma_x^2}), \quad \widehat{\tau}_{B_q} \sim \mathcal{N}(\tau_B, \frac{\widehat{\sigma_z^2}}{m\sigma_x^2}), \quad (16)$$

$$\widehat{\tau}_{A_p} \sim \mathcal{N}(\tau_A, \frac{\widehat{\sigma}_{z'}^2}{m\sigma_x^2}), \quad \widehat{\tau}_{B_p} \sim \mathcal{N}(\tau_B, \frac{\widehat{\sigma}_{z'}^2}{m\sigma_x^2}), \quad (17)$$

$$\frac{m\widehat{\sigma}_z^2}{\sigma_z^2} \sim \chi^2(m-2), \quad (18)$$

$$\frac{m\widehat{\sigma}_{z'}^2}{\sigma_{z'}^2} \sim \chi^2(m-2). \quad (19)$$

Note here that, in particular, $\widehat{\tau}_{A_q}$ and $\widehat{\tau}_{A_p}$ can be calculated by Alice without the exchange of variables, and then their value can be communicated to Bob. For this reason, Alice can manipulate all her data to estimate τ_A. The same is true for Bob, with respect to τ_B. In fact, both of them can combine the two estimators from the conjugate quadratures into a single one through a linear optimization, based on the estimator variance

$$\widehat{\tau}_A = c\widehat{\tau}_{A_q} + (1-c)\widehat{\tau}_{A_p} \quad \widehat{\tau}_B = c\widehat{\tau}_{B_q} + (1-c)\widehat{\tau}_{B_p} \quad (20)$$

with

$$\begin{aligned} c &= \frac{\mathrm{Var}(\widehat{\tau}_{A_p})}{\mathrm{Var}(\widehat{\tau}_{A_q}) + \mathrm{Var}(\widehat{\tau_{Ap}})} \\ &= \frac{\mathrm{Var}(\widehat{\tau}_{B_p})}{\mathrm{Var}(\widehat{\tau}_{B_q}) + \mathrm{Var}(\widehat{\tau}_{B_p})} = \frac{\widehat{\sigma}_{z'}^2}{\widehat{\sigma}_z^2 + \widehat{\sigma}_{z'}^2}. \end{aligned} \quad (21)$$

These new estimators have variance $c\frac{\widehat{\sigma}_z^2}{N\sigma_x^2}$. Therefore, one obtains

$$\widehat{T}_A = (2/\eta)\widehat{\tau}_A^2, \quad \widehat{T}_B = (2/\eta)\widehat{\tau}_B^2, \quad (22)$$

$$\widehat{\Xi} = \widehat{\sigma}_z - v_{\rm el} - 1, \quad \widehat{\Xi}' = \widehat{\sigma}_{z'} - v_{\rm el} - 1. \quad (23)$$

For large m, the distribution $\chi^2(m-2)$ can be considered to be Gaussian with variance $2m$. Next, based on

the previous considerations the parties derive confidence intervals for the channel parameters, such as

$$\tau_{A,B} \in [\widehat{\tau}_{A,B} - \Delta_\tau, \widehat{\tau}_{A,B} - \Delta_\tau], \tag{24}$$

$$\sigma^2_{z,z'} \in [\widehat{\sigma}^2_{z,z'} - \Delta_{z,z'}, \widehat{\sigma}^2_{z,z'} + \Delta_{z,z'}], \tag{25}$$

with

$$\Delta_\tau = w\sqrt{c\frac{\widehat{\sigma}^2_z}{N\sigma^2_x}}, \quad \Delta_{z,z'} = w\widehat{\sigma}^2_{z,z'}\sqrt{2/m}. \tag{26}$$

Finally, Alice and Bob calculate worst-case scenario values for the parameters T_A, T_B, Ξ, Ξ' obtained by the following formulas:

$$T_{Am} = \frac{(\widehat{\tau}_A - \Delta_\tau)^2}{(\eta/2)}, \quad T_{Bm} = \frac{(\widehat{\tau}_B - \Delta_\tau)^2}{(\eta/2)}, \tag{27}$$

$$\Xi_m = \widehat{\Xi} + \Delta_z, \qquad \Xi'_m = \widehat{\Xi}' + \Delta_{z'}. \tag{28}$$

III. COMPACT FORMULAS FOR WORST-CASE SCENARIO VALUES OF THE CALIBRATED ATTACK

Here we provide a compact form for the worst-case scenario values of the channel parameters

$$[T_A]_{\mathrm{wc}} = T_A D(T_A) \tag{29}$$

$$[T_B]_{\mathrm{wc}} = T_B D(T_B) \tag{30}$$

$$[\sigma^2_z]_{\mathrm{wc}} = \sigma^2_z d \tag{31}$$

where

$$D(T_A) = 1 - 2\sqrt{2}wm^{-1/2}\sqrt{Y_A}\sqrt{1 + \frac{\sigma^2_z}{\eta_{\mathrm{eff}} Y_A T_A \sigma^2_A}}, \tag{32}$$

$$D(T_B) = 1 - 2\sqrt{2}wm^{-1/2}\sqrt{Y_B}\sqrt{1 + \frac{\sigma^2_z}{\eta_{\mathrm{eff}} Y_B T_B \sigma^2_B}}, \tag{33}$$

$$Y_A = 1 + \frac{1}{2}\frac{T_B\sigma^2_B}{T_A\sigma^2_A}, \tag{34}$$

$$Y_B = 1 + \frac{1}{2}\frac{T_A\sigma^2_A}{T_B\sigma^2_B}. \tag{35}$$

IV. UNCALIBRATED ATTACK

In this section, we present the calculations for an uncalibrated attack (see Fig. 2 (a)); we then recover the calibrated attack described in the main text as a special case.

The input-output dependency is described by the following equations of the relay variables Q_R, P_R and the variables of the parties Q_A, P_A, Q_B, P_B:

$$Q_R = \tau_B Q_B - \tau_A Q_A + Q_z, \tag{36}$$

$$P_R = \tau_B P_B + \tau_A P_A + P_z, \tag{37}$$

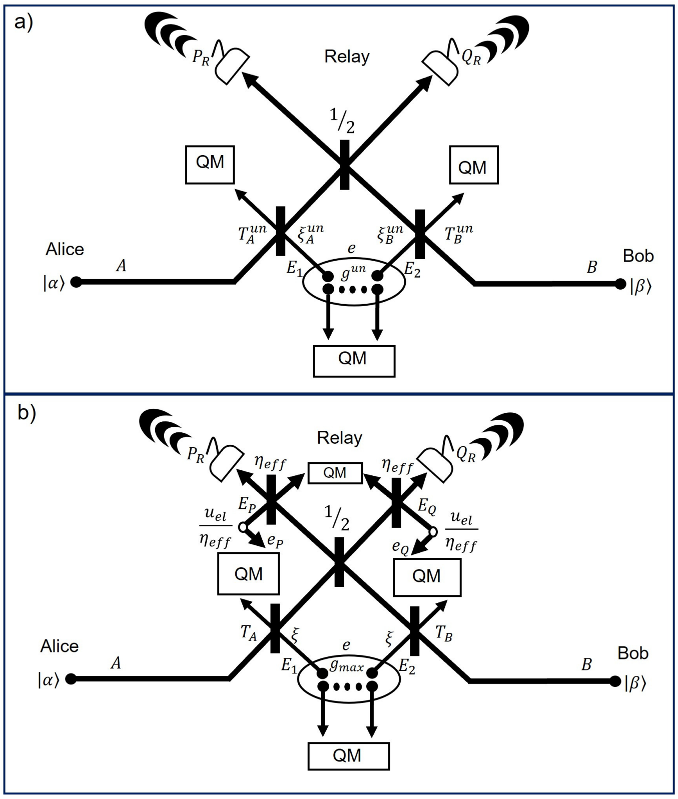

FIG. 2: In the first panel (a) we describe the uncalibrated attack with channel parameters T_A^{un}, T_B^{un}, ξ_A^{un}, ξ_B^{un}. By replacing with Eq. (52) till (56), we recover as a special case the calibrated attack in the second panel (b) where Eve has two extra TMSV states (in the modes $E_P e_P$ and $E_Q e_Q$). These are characterized by excess noise $u_{\mathrm{el}}/\eta_{\mathrm{eff}}$ and interact with the signal modes via two beam splitters with transmissivity η_{eff}.

where τ_A and τ_B are rescaling parameters connected to the overall attenuation via

$$\tau_A = \sqrt{T_A^{\mathrm{un}}/2}, \tag{38}$$

$$\tau_B = \sqrt{T_B^{\mathrm{un}}/2}, \tag{39}$$

where T_A^{un} and T_B^{un} are the uncalibrated overall transmissivities of the two links. The noise variables Q_z and P_z have variance σ^2_z such that

$$(\sigma^{\mathrm{un}})^2_z = \Xi^{\mathrm{un}} + 1, \tag{40}$$

and

$$\Xi^{\mathrm{un}} = \frac{1}{2}\left(T_A^{\mathrm{un}}\xi_A^{\mathrm{un}} + T_B^{\mathrm{un}}\xi_B^{\mathrm{un}}\right) + g^{\mathrm{un}}\sqrt{(1 - T_A^{\mathrm{un}})(1 - T_B^{\mathrm{un}})} \tag{41}$$

with

$$g^{\mathrm{un}} \leq \max\{\sqrt{(\omega_A^{\mathrm{un}} - 1)(\omega_B^{\mathrm{un}} + 1)}, \sqrt{(\omega_B^{\mathrm{un}} - 1)(\omega_A^{\mathrm{un}} + 1)}\}, \tag{42}$$

with

$$\omega_A^{\rm un} = \frac{T_A^{\rm un}\xi_A^{\rm un}}{1-T_A^{\rm un}} + 1, \tag{43}$$

$$\omega_B^{\rm un} = \frac{T_B^{\rm un}\xi_B^{\rm un}}{1-T_B^{\rm un}} + 1. \tag{44}$$

The uncalibrated total and conditional CMs are given by

$$\mathbf{V}_{ab|\gamma}^{\rm un} = \begin{pmatrix} \zeta^{\rm un}\mathbf{I} & \eta^{\rm un}\mathbf{Z} \\ \eta^{\rm un}\mathbf{Z} & \theta^{\rm un}\mathbf{I} \end{pmatrix}, \tag{45}$$

and

$$\mathbf{V}_{a|\gamma\tilde{\beta}}^{\rm un} = \left(\zeta^{\rm un} - \frac{(\eta^{\rm un})^2}{\theta^{\rm un}+1}\right)\mathbf{I}, \tag{46}$$

where

$$\zeta^{\rm un} = 1+\sigma_A^2 - \frac{(T_A^{\rm un}/2)\sigma_A^2(\sigma_A^2+2)}{\frac{T_B^{\rm un}\sigma_B^2+T_A^{\rm un}\sigma_A^2}{2} + (\sigma_z^{\rm un})^2}, \tag{47}$$

$$\theta^{\rm un} = 1+\sigma_B^2 - \frac{(T_B^{\rm un}/2)\sigma_B^2(\sigma_B^2+2)}{\frac{T_B^{\rm un}\sigma_B^2+T_A^{\rm un}\sigma_A^2}{2} + (\sigma_z^{\rm un})^2}, \tag{48}$$

$$\eta^{\rm un} = \frac{(\sqrt{T_A^{\rm un}T_B^{\rm un}}/2)\sqrt{\sigma_A^2(\sigma_A^2+2)\sigma_B^2(\sigma_B^2+2)}}{\frac{T_B^{\rm un}\sigma_B^2+T_A^{\rm un}\sigma_A^2}{2} + (\sigma_z^{\rm un})^2}. \tag{49}$$

V. SPECIFICATION TO THE CALIBRATED ATTACK

Here we describe the calibrated excess noise and efficiency of the detectors as Eve tampering with the relay modes before measurement. In this attack, the CM of Eve's modes is given by [10]

$$\mathbf{V}_E = \mathbf{V}_{E_1E_2} \oplus \mathbf{V}_{E_qe_q} \oplus \mathbf{V}_{E_pe_p}, \tag{50}$$

where $\mathbf{V}_{E_1E_2}$ is given by [2, Eq.(3)] while modes E_q and E_p with excess noise $\frac{u_{\rm el}}{\eta_{\rm eff}}$ interact with the relay modes before the homodyne detection through beam splitters of transmissivity $\eta_{\rm eff}$ as it is illustrated in Fig 2 (b). In particular, by setting $\omega_{\rm el} = \frac{u_{\rm el}}{1-\eta_{\rm eff}}+1$, we obtain the CMs

$$\mathbf{V}_{E_pe_p} = \mathbf{V}_{E_qe_q} = \begin{pmatrix} \omega_{\rm el}\mathbf{I} & \sqrt{\omega_{\rm el}^2-1}\mathbf{Z} \\ \sqrt{\omega_{\rm el}^2-1}\mathbf{Z} & \omega_{\rm el}\mathbf{I}. \end{pmatrix} \tag{51}$$

One may recover the calibrated attack from the uncalibrated one (see Appendix IV), by setting

$$T_A^{\rm un} = \eta_{\rm eff}T_A, \tag{52}$$

$$T_B^{\rm un} = \eta_{\rm eff}T_B, \tag{53}$$

$$\xi_A^{\rm un} = \xi + \frac{u_{\rm el}}{\eta T_A}, \tag{54}$$

$$\xi_B^{\rm un} = \xi + \frac{u_{\rm el}}{\eta T_B}, \tag{55}$$

$$g^{\rm un} = \eta_{\rm eff}g_{\rm max}\frac{\sqrt{(1-T_A)(1-T_B)}}{\sqrt{(1-\eta_{\rm eff}T_A)(1-\eta_{\rm eff}T_B)}}. \tag{56}$$

In this way, we obtain that

$$\begin{aligned}(\sigma_z^{\rm un})^2 &= \frac{1}{2}(T_A^{\rm un}\xi_A^{\rm un} + T_B^{\rm un}\xi_B^{\rm un}) \\ &+g^{\rm un}\sqrt{(1-T_A^{\rm un})(1-T_B^{\rm un})}+1 \\ &= \frac{\eta_{\rm eff}}{2}(T_A\xi + T_B\xi) + u_{\rm el} + 1 \\ &+ \eta_{\rm eff}g\frac{\sqrt{(1-T_A)(1-T_B)}\sqrt{(1-\eta_{\rm eff}T_A)(1-\eta_{\rm eff}T_B)}}{\sqrt{(1-\eta_{\rm eff}T_A)(1-\eta_{\rm eff}T_B)}} \\ &=\sigma_z^2.\end{aligned} \tag{57}$$

Note that, with the previous replacements, the Eqs. (47), (48), and (49) become the same as [2, Eqs.(18), (19), and (20)] respectively.

The secret key rate can be calculated based only on the total and conditional CMs. Therefore, we obtain the uncalibrated asymptotic rate by the previous replacements that are connected with the calibrated attack.

When the calculations include PE, the worst-case scenario values are given by

$$[T_A^{\rm un}]_{\rm wc} = T_A^{\rm un}D^{\rm un}(T_A^{\rm un}) \tag{58}$$

$$[T_B^{\rm un}]_{\rm wc} = T_B^{\rm un}D^{\rm un}(T_B^{\rm un}) \tag{59}$$

$$[(\sigma_z^{\rm un})^2]_{\rm wc} = (\sigma_z^{\rm un})^2 d \tag{60}$$

where

$$D^{\rm un}(T_A^{\rm un}) = 1 - 2\sqrt{2}wm^{-1/2}\sqrt{Y_A^{\rm un}}\sqrt{1+\frac{(\sigma_z^{\rm un})^2}{Y_A^{\rm un}T_A^{\rm un}\sigma_A^2}} \tag{61}$$

$$D^{\rm un}(T_B^{\rm un}) = 1 - 2\sqrt{2}wm^{-1/2}\sqrt{Y_B^{\rm un}}\sqrt{1+\frac{(\sigma_z^{\rm un})^2}{Y_B^{\rm un}T_B^{\rm un}\sigma_B^2}} \tag{62}$$

$$d = (1+\sqrt{2}wm^{-1/2}) \tag{63}$$

$$Y_A^{\rm un} = 1 + \frac{1}{2}\frac{T_B^{\rm un}\sigma_B^2}{T_A^{\rm un}\sigma_A^2} \tag{64}$$

$$Y_B^{\rm un} = 1 + \frac{1}{2}\frac{T_A^{\rm un}\sigma_A^2}{T_B^{\rm un}\sigma_B^2} \tag{65}$$

Therefore the CM entries after PE become

$$\zeta_{\rm PE}^{\rm un} = 1+\sigma_A^2 - \frac{\frac{[T_A^{\rm un}]_{\rm wc}}{2}\sigma_A^2(\sigma_A^2+2)}{\frac{[T_A^{\rm un}]_{\rm wc}\sigma_B^2+[T_B^{\rm un}]_{\rm wc}\sigma_A^2}{2} + [(\sigma_z^{\rm un})^2]_{\rm wc}}, \tag{66}$$

$$\theta_{\rm PE}^{\rm un} = 1+\sigma_B^2 - \frac{\frac{[T_B^{\rm un}]_{\rm wc}}{2}\sigma_B^2(\sigma_B^2+2)}{\frac{[T_A^{\rm un}]_{\rm wc}\sigma_B^2+[T_B^{\rm un}]_{\rm wc}\sigma_A^2}{2} + [(\sigma_z^{\rm un})^2]_{\rm wc}}, \tag{67}$$

$$\eta_{\rm PE}^{\rm un} = \frac{\frac{\sqrt{+[T_A^{\rm un}]_{\rm wc}+[T_B^{\rm un}]_{\rm wc}}}{2}\sqrt{\sigma_A^2(\sigma_A^2+2)\sigma_B^2(\sigma_B^2+2)}}{\frac{[T_A^{\rm un}]_{\rm wc}\sigma_B^2+[T_B^{\rm un}]_{\rm wc}\sigma_A^2}{2} + [(\sigma_z^{\rm un})^2]_{\rm wc}}. \tag{68}$$

After replacing from Eq. (58), (59), and (60), we obtain

$$\zeta_{\rm PE}^{\rm un} = 1+\sigma_A^2 - \frac{\frac{T_A^{\rm un}D^{\rm un}(T_A^{\rm un})}{2}\sigma_A^2(\sigma_A^2+2)}{\frac{T_B^{\rm un}D^{\rm un}(T_B^{\rm un})\sigma_B^2+T_A^{\rm un}D^{\rm un}(T_A^{\rm un})\sigma_A^2}{2}+d(\sigma_z^{\rm un})^2}, \tag{69}$$

$$\theta_{\rm PE}^{\rm un} = 1+\sigma_B^2 - \frac{\frac{T_B^{\rm un}D^{\rm un}(T_B^{\rm un})}{2}\sigma_B^2(\sigma_B^2+2)}{\frac{T_B^{\rm un}D^{\rm un}(T_B^{\rm un})\sigma_B^2+T_A^{\rm un}D^{\rm un}(T_A^{\rm un})\sigma_A^2}{2}+d(\sigma_z^{\rm un})^2}, \tag{70}$$

$$\eta_{\rm PE}^{\rm un} = \frac{\frac{\sqrt{T_A^{\rm un}D^{\rm un}(T_A^{\rm un})T_B^{\rm un}D^{\rm un}(T_B^{\rm un})}}{2}\sqrt{\sigma_A^2(\sigma_A^2+2)\sigma_B^2(\sigma_B^2+2)}}{\frac{T_B^{\rm un}D^{\rm un}(T_B^{\rm un})\sigma_B^2+T_A^{\rm un}D^{\rm un}(T_A^{\rm un})\sigma_A^2}{2}+d(\sigma_z^{\rm un})^2}. \tag{71}$$

One then may observe that

$$Y_A^{\rm un} = Y_A, \tag{72}$$
$$Y_B^{\rm un} = Y_B, \tag{73}$$
$$D^{\rm un}(T_A^{\rm un}) = D^{\rm un}(\eta_{\rm eff}T_A) = 1-2\sqrt{2}wm^{-1/2}\sqrt{Y_A}\sqrt{1+\frac{\sigma_z^2}{\eta_{\rm eff}Y_AT_A\sigma_A^2}} = D(T_A), \tag{74}$$
$$D^{\rm un}(T_B^{\rm un}) = D^{\rm un}(\eta_{\rm eff}T_B) = 1-2\sqrt{2}wm^{-1/2}\sqrt{Y_B}\sqrt{1+\frac{\sigma_z^2}{\eta_{\rm eff}Y_BT_B\sigma_B^2}} = D(T_B), \tag{75}$$

where Y_A, Y_B, $D(T_A)$, $D(T_B)$ have been calculated in Appendix III. Taking the latter into consideration and replacing from Eq. (52), (53), and (57), we obtain

$$\zeta_{\rm PE}^{\rm un} = 1+\sigma_A^2 \tag{76}$$
$$- \frac{\frac{\eta_{\rm eff}}{2}T_AD(T_A)\sigma_A^2(\sigma_A^2+2)}{\eta_{\rm eff}\frac{T_BD(T_B)\sigma_B^2+T_AD^{\rm cal}(T_A)\sigma_A^2}{2}+d\sigma_z^2}, \tag{77}$$
$$\theta_{\rm PE}^{\rm un} = 1+\sigma_B^2 \tag{78}$$
$$- \frac{\frac{\eta_{\rm eff}}{2}T_BD(T_B)\sigma_B^2(\sigma_B^2+2)}{\eta_{\rm eff}\frac{T_BD(T_B)\sigma_B^2+T_AD^{\rm cal}(T_A)\sigma_A^2}{2}+d\sigma_z^2}, \tag{79}$$
$$\eta_{\rm PE}^{\rm un} = \frac{\frac{\eta_{\rm eff}\sqrt{T_AD(T_A)T_BD(T_B)}}{2}\sqrt{\sigma_A^2(\sigma_A^2+2)\sigma_B^2(\sigma_B^2+2)}}{\eta_{\rm eff}\frac{T_BD(T_B)\sigma_B^2+T_AD(T_A)\sigma_A^2}{2}+d\sigma_z^2}. \tag{80}$$

Finally, from the equations in Appendix III, we obtain

$$\zeta_{\rm PE}^{\rm un} = 1+\sigma_A^2 - \frac{\frac{\eta_{\rm eff}}{2}[T_A]_{\rm wc}\sigma_A^2(\sigma_A^2+2)}{\eta_{\rm eff}\frac{[T_B]_{\rm wc}\sigma_B^2+[T_A]_{\rm wc}\sigma_A^2}{2}+[\sigma_z^2]_{\rm wc}} = \zeta_{\rm PE} \tag{81}$$

$$\theta_{\rm PE}^{\rm un} = 1+\sigma_B^2 - \frac{\frac{\eta_{\rm eff}}{2}[T_B]_{\rm wc}\sigma_B^2(\sigma_B^2+2)}{\eta_{\rm eff}\frac{[T_B]_{\rm wc}\sigma_B^2+[T_A]_{\rm wc}\sigma_A^2}{2}+[\sigma_z^2]_{\rm wc}} = \theta_{\rm PE} \tag{82}$$

$$\eta_{\rm PE}^{\rm un} = \frac{\frac{\eta_{\rm eff}\sqrt{[T_A]_{\rm wc}[T_B]_{\rm wc}}}{2}\sqrt{\sigma_A^2(\sigma_A^2+2)\sigma_B^2(\sigma_B^2+2)}}{\eta_{\rm eff}\frac{[T_B]_{\rm wc}\sigma_B^2+[T_A]_{\rm wc}\sigma_A^2}{2}+[\sigma_z^2]_{\rm wc}} = \eta^{\rm cal}. \tag{83}$$

This is the same covariance matrix for the calibrated attack assuming worst-case values from the PE step.

VI. RATE COMPARISON WITH PREVIOUS WORK

Here, we compare the composable rate established in this work with the rate stated in previous work by some of us [5]. There the calculations assumed a mathematical conjecture [5, Remark 5].

Definition VI.1 *A (trace-distance) ball of density operators around a density operator ρ is defined as*

$$\mathcal{B}_D^\epsilon(\rho) := \{\rho' : {\rm Tr}\,\rho' = 1,\ D(\rho',\rho)\le\epsilon\} \tag{84}$$

where $D(\rho',\rho)$ is the trace distance between the operators ρ' and ρ as defined in [6, Eq. (G24)].

Let us assume now a classical-quantum (CQ) state ρ_{XB}, where system X is classical. One may write

$$\rho_{XB} = \sum p_X(x)|x\rangle\langle x|_X\otimes\rho_{B|X=x}, \tag{85}$$

where $p_X(x)$ is the classical distribution of a variable X which takes values $x\in\mathcal{X}$, and $\rho_{B|X=x}$ is the conditional state of system B. Then the following conjecture was suggested in [5, Remark 5]:

Conjecture VI.1 *For a CQ normalized state (density operator) ρ_{XB} there is (always) a CQ state $\rho^\star_{XB}$ such that $D(\rho^\star,\rho)\le\epsilon$ (i.e., $\rho^\star\in\mathcal{B}_D^\epsilon(\rho)$) and*

$$H^\epsilon_{min}(X|B)_\rho = H_{min}(X|B)_{\rho_\star}, \tag{86}$$

where $H^\epsilon_{min}(X|B)_\rho$ is the smooth min-entropy of ρ and $H_{min}(X|B)_{\rho_\star}$ is the min-entropy of $\rho_\star$.

This conjecture led to the following terms in the key rate of Ref. [5]:

$$\widetilde{\Delta}_{\rm AEP} = 4(2p+1)\sqrt{\log_2(18/4p_{\rm EC}^2\epsilon_{\rm s}^2)}, \tag{87}$$
$$\widetilde{\Theta} = \log_2[p_{\rm EC}(1-\frac{2}{3}\varepsilon_{\rm s})]+2\log_2(2\epsilon_{\rm h}). \tag{88}$$

While this conjecture still remains unproven, a rigorous statement can be made for CQ states. To make use of

this statement, we need to relax two assumptions. We first need to consider sub-normalized states, i.e., states σ with $\mathrm{Tr}(\sigma) \leq 1$ and, secondly, define balls based on the purified distance instead of the trace distance.

Definition VI.2 *A (purified-distance) ball of sub-normalized states around a sub-normalized state σ is defined as*

$$\mathcal{B}_P^{\epsilon}(\sigma) := \{\sigma' : \mathrm{Tr}\,\sigma' \leq 1, P(\sigma', \sigma) \leq \epsilon \leq 1\} \quad (89)$$

where $P(\sigma', \sigma)$ is the purified distance between the operators σ' and σ as defined in [6, Appendix G.2].

Then we have the following proposition proven in Ref. [7, Prop. 5.8]:

Proposition VI.2 *For a sub-normalized state σ_{XB} that is classical on X there exists a state $\sigma^{\star}_{XB} \in \mathcal{B}_P^{\epsilon}(\sigma)$ that is classical on X such that*

$$H^{\epsilon}_{min}(X|B)_{\sigma} = H_{min}(X|B)_{\sigma^{\star}} \quad (90)$$

for $\epsilon \geq 0$.

Note that we use a simplified version of the original version of the proposition in Ref. [7]. According to this proposition, we use bounds for the CQ states based on the purified distance which leads to a different composable term Δ_{AEP} (see [2, Eq. (83)]). Besides this, taking into consideration the normalization of the sub-normalized states (e.g., see [6, Eqs. (G30), (G34), (G35)]) we obtain a difference in Θ as well (see [2, Eq. (84)]). In particular, one may see the very different scaling in ϵ_{s}, i.e., $\epsilon_{\mathrm{s}} \to \epsilon_{\mathrm{s}}^2$, so that

$$\widetilde{\Delta}_{\mathrm{AEP}}(\epsilon, p) \propto \sqrt{\log_2(1/\epsilon_{\mathrm{s}}^2)} \quad (91)$$

$$\Delta_{\mathrm{AEP}}(\epsilon, p) \propto \sqrt{\log_2(1/\epsilon_{\mathrm{s}}^4)} \quad (92)$$

In addition to the above, the first part of the Θ terms is different so that:

$$\tilde{\Theta} \propto \log_2\left[p_{\mathrm{EC}}(1 - \frac{2}{3}\epsilon_{\mathrm{s}})\right] \quad (93)$$

$$\Theta \propto \log_2\left[p_{\mathrm{EC}}(1 - \frac{1}{3}\epsilon_{\mathrm{s}}^2)\right] \quad (94)$$

One should also note two different prefactors in the delta quantities of [2, Eq. (83)] and (87), namely,

$$F(p) = 4\log_2(2^p + 2) \text{ and } \widetilde{F}(p) = 4(2p + 1). \quad (95)$$

This difference comes from an extra approximation in Ref. [5].

One may also observe that the second term of Θ is also different from $\widetilde{\Theta}$ (there is a $\sqrt{2}$ as a factor inside the logarithm instead of a factor 2). This is due to a bound used in Ref. [5] with a different direction. Therefore, our rate has very different composable terms (Δ_{AEP} and Θ) and dependence on the security parameters $\epsilon_{\mathrm{h,s}}$ with respect to the rate from Ref. [5]. An example of the practical differences between the two rates in terms of performance is illustrated in Fig. 1. For the regime explored, the formula from Ref. [5] turns out to be way more pessimistic than our revised rate. This over-pessimistic feature turns out to be beneficial as it would enable one to use the rate from Ref. [5] as a lower bound.

VII. MODULAR NETWORK FOR QUANTUM CONFERENCING

Here, we also revise the conferencing key rate of the modular network presented in Ref. [8]. Let us consider the discretized versions b_i and b_j of the variables x_i and y_j after the correction of β_i and β_j with γ_i and γ_j that correspond to the i_{th} and j_{th} users respectively (see Ref. [8] for this notation). Then the state before EC will be given by a tensor product $\rho^{\otimes n}$ of states

$$\rho = \sum_{b_i, b_j} p(b_i, b_j)|b_i\rangle\langle b_i| \otimes |b_j\rangle\langle b_j| \otimes \rho_{\mathbf{E}\boldsymbol{\gamma}}(b_i, b_j), \quad (96)$$

where $\mathbf{E}$ is Eve's system and $\boldsymbol{\gamma}$ is the vector of outcomes of the generalized Bell measurement. The EC procedure can be simulated by a projection Π_{cor} to correctable sequences $\tilde{b}_i$ with success probability

$$p_{\mathrm{EC}} = \mathrm{Tr}(\Pi_{\mathrm{cor}}\rho^{\otimes n}), \quad (97)$$

and the state after EC is given by

$$\tilde{\rho}^n = p_{\mathrm{EC}}^{-1}\Pi_{\mathrm{cor}}\rho^{\otimes n}\Pi_{\mathrm{cor}}. \quad (98)$$

According to the direct leftover hash lemma, one may bound the secret key bits derived after PA with

$$s_n \geq H^{\varepsilon_{\mathrm{s}}}_{\min}(b_j|\mathbf{E}^n\boldsymbol{\gamma}^n)_{\tilde{\rho}^n} + 2\log_2\sqrt{2}\varepsilon_{\mathrm{h}} - \mathrm{leak}_{\mathrm{EC}}. \quad (99)$$

The state after PA $\bar{\rho}^n$ has a distance from the ideal state

$$\rho_{\mathrm{id}} = 2^{-s_n}\sum_{z=0}^{2^{s_n}-1}|z\rangle\langle z| \otimes |z\rangle\langle z| \otimes \rho_{\mathbf{E}^n\boldsymbol{\gamma}^n} \quad (100)$$

that is equal to

$$p_{\mathrm{EC}}D(\bar{\rho}^n, \rho_{\mathrm{id}}) \leq \epsilon_{\mathrm{sec}}. \quad (101)$$

Here $H^{\varepsilon_{\mathrm{s}}}_{\min}(b_j|\mathbf{E}^n\boldsymbol{\gamma}^n)_{\tilde{\rho}^n}$ is calculated based on the state after EC which, according to the triangular inequality, has a distance

$$p_{\mathrm{EC}}D(\tilde{\rho}^n, \rho_{\mathrm{id}}) \leq \epsilon_{\mathrm{sec}} + \epsilon_{\mathrm{cor}} \quad (102)$$

with

$$p_{\mathrm{EC}}\mathrm{Prob}(b_i \neq b_j) \leq \epsilon_{\mathrm{cor}} \quad (103)$$

after the verification step.

Then we may connect the previous result with the smooth min-entropy of the state before EC, which is in

a tensor product form and thus subject to the AEP approximation. We have that

$$s_n \geq H_{\min}^{p_{\rm EC}\epsilon_{\rm s}^2/3}(b_j|\mathbf{E}^n\boldsymbol{\gamma}^n)_{\rho^{\otimes n}} + 2\log_2\sqrt{2}\epsilon_{\rm h} - {\rm leak}_{\rm EC} + \log_2[p_{\rm EC}(1-\epsilon_{\rm s}^2/3)], \tag{104}$$

where we use Eq. (G9) of Ref. [6].

After the AEP approximation, we derive

$$s_n \geq nH(b_j|\mathbf{E}\boldsymbol{\gamma})_\rho - \sqrt{n}\Delta_{\rm AEP}(p_{\rm EC}\epsilon_{\rm s}^2/3, d) + 2\log_2\sqrt{2}\epsilon_{\rm h} - {\rm leak}_{\rm EC} + \log_2[p_{\rm EC}(1-\epsilon_{\rm s}^2/3)] \tag{105}$$

with (see Ref. [9, Eq. (66)])

$$\Delta_{\rm AEP}(\epsilon_{\rm s}, p) = 4\log_2(2^p+2)\sqrt{\log_2(2/\epsilon_{\rm s}^2)}, \tag{106}$$

where p is the number of bits in the discretization of b_i and $H(b_j|\mathbf{E}\boldsymbol{\gamma})_\rho$ is the conditional von Neumann entropy calculated with respect to ρ.

Then, we have

$$H(b_j|\mathbf{E}\boldsymbol{\gamma})_\rho = H(b_j|\boldsymbol{\gamma}) - \chi(b_j:\mathbf{E}|\boldsymbol{\gamma}), \tag{107}$$

and $H(b_j|\boldsymbol{\gamma}) = H(b_j)$. In turn, we may apply the following replacement

$$\xi I(\beta_i:\beta_j|\boldsymbol{\gamma}) = H(b_j) - n^{-1}{\rm leak}_{\rm EC}. \tag{108}$$

Similar to [2, Eq. (40)], we then have

$$\chi(b_j:\mathbf{E}|\boldsymbol{\gamma}) = \chi(\beta_j:\mathbf{E}|\boldsymbol{\gamma}). \tag{109}$$

Hence, by dividing Eq. (105) by the number of channel uses N, we can write the secret key rate

$$R = \frac{np_{\rm EC}}{N}\left[R_{\rm mod} - \frac{\Delta_{\rm AEP}}{\sqrt{n}} + \frac{\Theta}{n}\right], \tag{110}$$

where

$$R_{\rm mod} = \xi I(\beta_i:\beta_j|\boldsymbol{\gamma}) - \chi(\beta_j:\mathbf{E}|\boldsymbol{\gamma}). \tag{111}$$

The rate in Eq. (110) is a revision of the one stated in Eq. (7) of Ref. [8]. Note that $R_{\rm mod}$ also needs to be replaced by the corresponding expression in terms of the worst-case estimators. Overall the epsilon security of the protocol will be

$$\epsilon = \epsilon_{\rm cor} + \epsilon_{\rm h} + \epsilon_{\rm s} + p_{\rm EC}\tilde{\epsilon}_{\rm PE} \tag{112}$$

where $\tilde{\epsilon}_{\rm PE}$ will be dependent on the number of different estimators that participate in PE.

[1] T. M Cover and J. A. Thomas, “Elements of information theory,” (Wiley, 2012).

[2] P. Papanastasiou, A. G. Mountogiannakis, and S. Pirandola, "Composable security of CV-MDI-QKD: Secret key rate and data processing"

[3] P. Papanastasiou, C. Ottaviani, and S. Pirandola, “Finite-size analysis of measurement-device-independent quantum cryptography with continuous variables,” Phys. Rev. A **96**, 042332 (2017).

[4] For instance, see: Dmitry Panchenko, 18.650 Statistics for Applications, Fall 2006, MIT.

[5] C. Lupo, C. Ottaviani, P. Papanastasiou, and S. Pirandola, “Continuous-variable measurement-device-independent quantum key distribution: Composable security against coherent attacks,” Phys. Rev. A **97**, 052327 (2018).

[6] S. Pirandola, “Limits and Security of Free-Space Quantum Communications,” Phys. Rev. Research **3**, 013279 (2021).

[7] M. Tomamichel, “A Framework for Non-Asymptotic Quantum Information Theory” (PhD thesis, Zurich 2005).

[8] C. Ottaviani, C. Lupo, R. Laurenza, and S. Pirandola, “Modular network for high-rate quantum conferencing,” Communications Physics **2**, 118 (2019).

[9] S. Pirandola, “Composable security for continuous-variable quantum key distribution: Trust levels and practical key rates in wired and wireless,” Phys. Rev. Research **3**, 043014 (2021).

[10] Note that we base our final calculation of the rate on Alice’s and Bob’s systems (the latter is purified by Eve). Therefore, we may have a more intricate description for Eve which may add extra variables in the total CM but does not change the structure of the calculations
